# Phage Display Panning on Silica: Optimization of Elution Conditions for Selection of Strong Binders

**DOI:** 10.1021/acs.langmuir.4c01108

**Published:** 2024-07-17

**Authors:** Veeranjaneyulu Thota, Valeria Puddu, Carole C. Perry

**Affiliations:** Interdisciplinary Biomedical Research Centre, School of Science and Technology, Nottingham Trent University, Clifton Lane, Nottingham NG11 8NS, U.K.

## Abstract

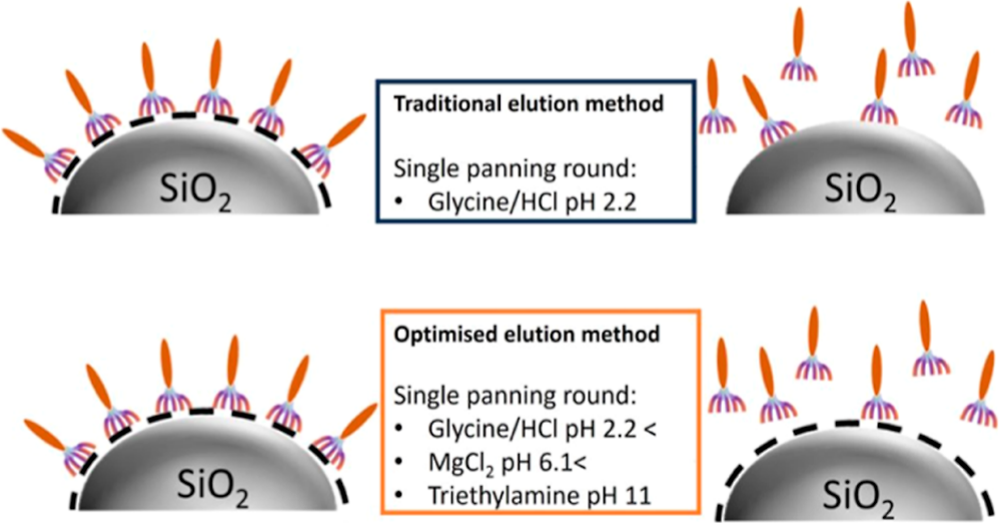

Phage display panning is a powerful tool to select strong peptide binders to a given target, and when applied to inorganic materials (e.g., silica) as a target, it provides information on binding events and molecular recognition at the peptide–mineral interface. The panning process has limitations with the phage chemical elution being affected by bias toward positively charged binders, resulting in the potential loss of information on binder diversity; the presence of fast growing phages with an intrinsic growth advantage; and the presence of false positives from target unrelated peptides. To overcome some of these limitations, we developed a panning approach based on the sequential use of different eluents (Gly-HCl, pH-2.2; MgCl_2_, pH-6.1; and TEA, pH-11.0), or pH conditions (Gly-HCl 2.2 < pH < 11.0) that allows the identification of a diverse and comprehensive pool of strong binders. We have assessed and tested the authenticity of the identified silica binders via a complementary experimental (in vivo phage recovery rates and TEM imaging) and bioinformatics approach. We provide experimental evidence of the nonspecificity of the Gly-HCl eluent as typically used. Using a fluorimetric assay, we investigate in vitro binding of two peptides that differ by pI–S4 (HYIDFRW, pI 7.80) and S5 (YSLKQYQ, pI 9.44)—modified at the C terminal with an amide group to simulate net charges in the phage display system, confirming the vital role of electrostatic interactions as driving binding forces in the phage panning process. The presented optimized phage panning approach provides an opportunity to match known surface interactions at play with suitable elution conditions; to select only sequences relevant to a particular interfacial system. The approach has the potential to open up avenues to design interfacial systems to advance our understanding of peptide-assisted mineral growth, among other possibilities.

## Introduction

Phage panning is an iterative in vivo screening process that uses phage display libraries consisting of a large population (10^9^–10^10^) to select the affinity of peptides toward a given target. The screening technique was initially developed to study protein–protein molecular recognition; in recent years, its application has widened to include inorganic materials as targets, becoming a convenient tool in the identification of mineral-binding peptides,^1,2^ and in the advancement of our understanding of the processes occurring at the organic–inorganic interface.^3^ Its application in the field of molecular biomimetics, nano- and biotechnology means that it is now possible to produce novel materials using biological or bioinspired templates or using them as linkers to create hybrids with unique mechanical, electronic, photonic, or magnetic properties.^4,5^

Phage panning consists of several steps, starting with incubation at neutral pH of the initial phage library with the desired target followed by a series of washings to remove unbound phage. A low pH eluent (Gly-HCl at pH 2.2) is typically used to recover strongly bound phages, thus obtaining a pool of phages that is then amplified before undergoing another panning cycle. Panning rounds between three and five are performed before sequencing, to ensure strong binder enrichment throughout the process.^6^

Biopanning results are known to be affected by false positives in the form of target unrelated peptides (TUPs). TUPs are peptides abundant in the final output not because of their specificity to the target but because of a propagation advantage during the amplification step (propagation-related TUPs), or because of background binding attributed to other constituents of the panning system like plastic, contaminants, or capturing agents (selection-related TUPs).^7^ Bioinformatic analyses based on specific databases for phage display data, such as the biopanning data bank (BDB), are proving important to the biopanning community to clean their panning results of false positives from TUPs.^8^

An increasing number of mineral-binding peptides have been isolated on inorganic surfaces, such as silica, using phage panning. Screening on mineral targets appear to be dependent on a variety of physical (e.g., morphology, size, crystal phase, and orientation) and chemical (hydrogen bonding, polarity, and charge effects) properties of the interface under study.^9^ For example, a range of sequences with heterogeneous composition and properties, rather than a consensus sequence, have been typically identified as strong silica binders based on phage display results. Differences in the sequence composition of the identified peptides have been ascribed to the complex structure of the inorganic surface, degree of surface ionization, and minor differences in size of the silica nanoparticles used as targets.^9−12^ The complexity of the possible interactions occurring at the mineral–peptide interface can also affect how a given inorganic target responds to the incubation and elution conditions during the phage panning process. In our previous studies,^9,12^ we evidenced how pH affects the binding behavior in vitro of strong silica binders selected by phage panning (S1, S2, and S3). Peptide binding studies and computational data show that binding behavior is directly dependent on the peptide sequence and predicted charge specific interactions with the silica surface. These in vitro binding results strongly suggest that binding and elution conditions used in the conventional phage panning protocol (i.e., neutral pH for binding and acidic pH using Gly-HCl pH 2.2 for elution) may suffer from an intrinsic bias which favors the elution of positively charged sequences, leaving behind a pool of strong binding sequences that attach on the silica surface via weaker non-Coulombic interactions which are not efficiently disrupted by the drop in pH used in the elution step. Alternative elution strategies, based on pH stepwise elution have been reported for panning against biological targets,^13^ however use of Gly-HCl pH 2.2 is still dominant and to the best of our knowledge more or less exclusively used for inorganic targets, potentially leaving strong binders based on non-Coulombic interactions undetected.

In this study, we explore how different elution conditions, acidic, neutral, and basic pH, affect sequence selection during the panning process. We then use these results to inform the design of an innovative and sustainable multi eluent approach that uses different eluents sequentially, leading to the isolation of a comprehensive pool of strong binders from a single phage panning experiment. Throughout this work, we combine the experimental data (panning and phage recovery rates) and bioinformatic tools to exclude TUPs and develop a reliable protocol for the comprehensive selection of strong binding phage clones. Furthermore, we selected two peptide sequences, S4 and S5, to confirm in vitro their binding properties on silica. Binding affinities are typically evaluated using synthetic peptides; however, it is known that in phage display, the peptide sequence is fused to the phage via the C-terminus leaving the N-terminus available for interactions. This means that during phage panning, the overall charge of the binding sequence is different than that in the confirmation experiments. To bridge the gap between binding during phage panning and using synthetic sequences, we perform an amide modification of the peptides C-terminus to better mimic peptide presentation during phage display and study via fluorimetric assay and TEM imaging how the extent of binding and interfacial forces involved are affected by the amide modification.

## Experimental Section

**Scheme 1:**
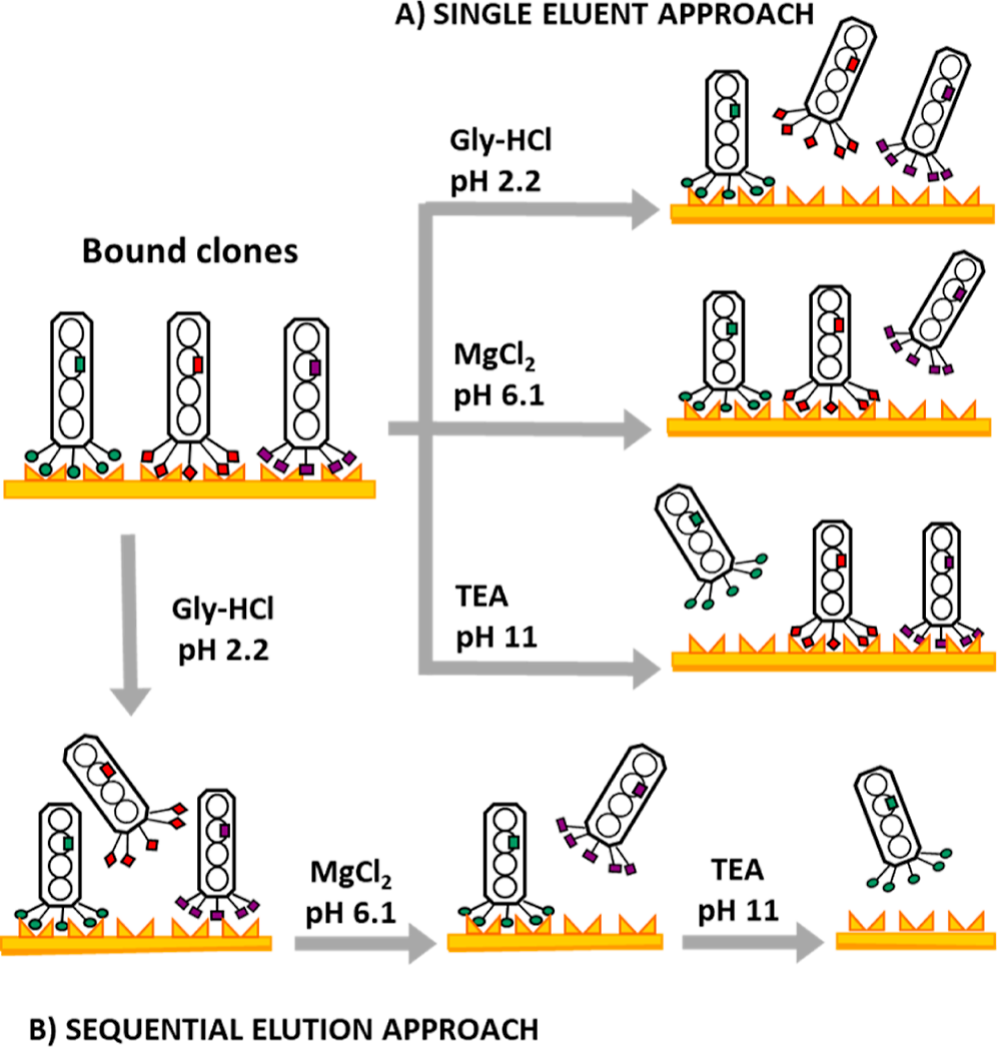
Representation of Elution Approaches Used in This Study: Single Eluent (a), and Sequential Elution (b), The Different Shaped “Ends” to the Displayed Peptide Indicate Different Sequences

Full experimental details on phage panning, binding studies, and bioinformatic tools used are reported in the Supporting Information. For phage panning experiments, briefly, a Ph.D.7 phage display library (NEC, batch 0211212) was used. For the entire study, the same batch of amorphous silica nanoparticles of size 82 nm and previously characterized^9−11^ was used as a target to avoid any inconsistencies that might occur due to differences in the silica surface properties. Throughout our panning experiments, we avoided using polystyrene to avoid selection of polystyrene-binding phages.^7^ Panning was carried out according to the peptide library manufacturer’s instructions, with modification to elution and neutralization conditions, leading to three panning protocols (Table 1). The two conditions used for elution were as follows: conventional single eluent approach, where a single eluent is used in multiple rounds of panning (up to three); and sequential elution approach, where multiple eluents and pH are used sequentially in a single round of panning (Scheme 1). The eluents used were as follows: Gly-HCl (pH 2.2), MgCl_2_ (pH 6.1), and triethylamine (TEA) (pH 11). The single eluent approach was used in what we describe as conventional panning and conventional repanning protocols; while the sequential elution approach was used in our optimized panning protocol. The optimized protocol includes two strategies, the first used different eluent types (i.e., Gly-HCl, MgCl_2_, and TEA), while the second used Gly-HCl at different pH values (2.2, 7, and 11). In both repanning and optimized protocols, the initial phage library is a mix of phage obtained by combination of amplified phage from round 2 of several conventional panning experiments (Table 1).

**Table 1:** Experimental Conditions Used in Conventional, Repanning, and Optimized Panning Procedures^a^

phage panning protocol	library lot	elution approach	eluent	molarity M	pH	screened round
conventional panning	lot 1 (0211212)	single eluent	Gly-HCl	0.2	2.2	3
			MgCl_2_	4	6.1	3
			TEA	0.1	11.0	3
conventional repanning	mix of amplified phages* from Lot 1 (0211212)	single eluent	Gly-HCl	0.2	2.2	3
			TEA	0.1	11.0	4
optimized panning	mix of amplified phages* from Lot 1 (0211212)	elution strategy 1				
		sequential eluents	Gly-HCl	0.2	2.2	3
			MgCl_2_	4	6.1	3
			TEA	0.1	11.0	3
		elution strategy 2				
		sequential eluents	Gly-HCl	0.2	2.2	3
					7.0	3
					11.0	3

a Experiments were performed at separate times using phages from the same library (#E8100Slot 1 0211212). *The mix of amplified phages used in the repanning and optimized method was obtained by mixing 50 μL from each round 2 amplified eluate obtained from five separate traditional panning experiments performed with different eluents.

## Results and Discussion

In the conventional panning protocol, we explore the effect of three different eluents and pH conditions on the sequences eluted, typically after three rounds of panning. To obtain information about each elution condition, a separate panning experiment was necessary. In the optimized panning method, we used the three eluents sequentially in a single panning experiment, obtaining three eluates per panning round. All eluates were analyzed for peptide sequence similarity, frequency, and properties, complete lists of isolated sequences are available in the Supporting Information.

### Conventional Panning—Effect of Different Eluents

We used three different eluents with varying pH values: Gly-HCl (pH 2.2), MgCl_2_ (pH 6.1), and triethylamine (TEA) (pH 11). A total of 72 individual phage plaques were selected randomly from the third round of different panning experiments and analyzed by DNA sequencing (Table S1). Out of 72 individual phage plaques picked for sequence analysis, 45 plaques resulted from Gly-HCl, pH-2.2 elution, 14 from MgCl_2_, pH-6.1 elution, and 13 from TEA, pH-11 elution.

The use of each of the three eluents following the conventional elution protocol allowed isolation after three rounds of panning of distinct sequence populations with different pI characteristics (Table 2). Two panning experiments were performed using Gly-HCl (pH 2.2), the eluent typically used in phage panning protocols, particularly for the identification of peptides binding to minerals.^5,9^ The first experiment yielded the consensus sequence LPVRLDW, while the second experiment provided a wide pool of sequences of pI in the range 3–11, with sequence GASESYL being frequently isolated. The other two eluents, neutral MgCl_2_ and basic TEA, allowed the identification of smaller pools of sequences, where sequences show higher frequencies. Most of the peptides identified using MgCl_2_ and TEA were found at least once in the Gly-HCl eluate. It is notable that all of the sequences eluted by MgCl_2_ had a circumneutral pI.

**Table 2:** Frequently Observed Sequences Isolated Using Conventional and Optimized Panning Methods^a^

	conventional elution with eluents used in different panning experiments			optimized elution with three eluents used consecutively in a single panning experiment		
eluent	sequences at third round	frequency	pI^a^	sequences at third round	frequency	pI^a^
Gly-HCl (pH 2.2)	LPVRLDW^b^	25/25	6.85	TVNFKLY	8/30	9.67
	GASESYL	6/20	3.27	VSRDTPQ	3/30	6.78
	VSRDTPQ	3/20	6.85	GQSEKHL	2/30	7.82
	WQWPARV	2/20	11.06	YSLKQYQ*(N)	2/30	9.44
	NDLMNRA	1/20	6.85	YNGSANG (N)	2/30	5.97
	GQSEKHL	1/20	7.88	VENVHVR (N)	2/30	7.90
	ALQPQKH	1/30	10.13	FASRSDT (N)	2/30	6.85
	ETALIAA	1/30	3.27	LPVRLDW	1/30	6.78
	HYIDFRW*(R)	2/30	7.80			
	HVPRAMA (R)	1/30	11.06			
	LPVRLDW (R)	1/30	6.78			
MgCl_2_ (pH 6.1)	LPVRLDW	5/14	6.85	VSRDTPQ	12/30	6.78
	NDLMNRA	4/14	6.85	GQSEKHL	6/30	7.82
	GQSEKHL	2/14	7.88	TVNFKLY	1/30	9.67
	QLAVAPS	1/14	6.01	ELTPLPL	1/30	3.30
TEA (pH 11)	GQSEKHL	7/13	7.88	YSLKQYQ*(N)	12/30	9.44
	ELTPLPL	2/13	3.27	YSFKQYQ (N)	5/30	9.44
	QHMPQPR	1/13	11.06	YNGSANG (N)	3/30	5.97
	NDLMNRA	1/13	6.85			
	VSRDTPQ	1/13	6.85			
	KIAVIST	1/13	10.13			
	HYIDFRW*(R^$^)	3/30	7.80			
	SFPLSKY(R^$^)	3/30	9.67			

a (R) indicates new sequences identified in the repanning experiment. (N) indicates new sequences identified in the optimized elution. An asterisk (*) indicates sequences selected for in vitro binding studies/effect of the amide group at the C-terminus. ^$^ indicates sequences isolated at round 4 of panning. ^a^pI values were obtained from the Bachem peptide calculator (http://www.bachem.com/). ^b^ Phages from this experiment were not included in the amplified phage mix used in the repanning experiments.

As part of our method development, we validated the use of a “repanning” protocol, where a mixture of amplified phages from previously performed experiments on the same silica target is used as the initial phage pool. Binders were eluted using Gly-HCl or TEA (low and high pH elution conditions, respectively) at rounds 3–4 (Table 2), using different levels of wash stringency. This approach aims to check against the selection of false positives due to their panning limitation or amplification bias and to verify the overall quality of our panning. Sequencing results from 120 randomly selected phage plaques reproduced a similar population of silica binders, as previously identified in conventional panning (Table S2). Out of 120 phage clones, 102 phage clones previously identified were reproduced in the repanning experiment, indicating 85% similarity. Of these, four sequences (LPVRLDW, GQSEKHL, NDLMNRA, and VSRDTPQ) were isolated following elution at both low and high pH conditions in the repanning experiments. In addition, six new sequences were identified from 18 phage clones (15%). These include sequence HYIDFRW, isolated at both high and low pH conditions. Eluates from the experiment leading to the isolation of the consensus sequence LPVRLDW (Table 2) were not included in the starting mix of the amplified phage for repanning. The recurrence of phage clones, displaying the peptide LPVRLDW in this series of repanning experiments, suggests that the sequence isolation does not derive from propagation advantage, as further confirmed by bioinformatic tools.

### Optimized Panning: Tailoring Elution Conditions to Extract Specific Sequences

The effective identification of different sequences by varying elution conditions shown above underpins the design of an optimized elution protocol where the three elution conditions are used sequentially in a single panning round, making the entire process easier to perform and cost and time effective. A few studies have used a stepwise decrease of the pH of the eluent (from pH 5 to pH 2) in the third and final round of panning to improve the selection of high affinity binders on antibodies,^14,15^ to the best of our knowledge this approach has not been applied to phage panning on inorganic surfaces.

We evaluated two approaches: in the first approach, the three eluents were used consecutively at each elution step in the following order: first Gly-HCl (pH-2.2), second MgCl_2_ (pH-6.1), and third TEA, pH-11. By using the same three eluents sequentially in a single panning round, we expected to elute and recover silica binders that are bound to silica by both electrostatic and nonelectrostatic interactions in one round. In the second approach, to compare whether the same effect could be realized by simply shifting the pH, Gly-HCl was used at different pH values (pH-2.2, pH-7, and pH-11) sequentially.

The same mixture of amplified phages used in the repanning experiments was used as the starting phage library. For each approach, we randomly selected 30 phage clones for each eluent used. Panning results for the two approaches were similar; we present and discuss results for the elution approach using several types of eluents here (Table 2), while results for Gly-HCl at different pH values are reported in the Supporting Information (Table S1–S3). Sequence analysis shows that stepwise elution with Gly-HCl and MgCl_2_ reproduced some binders already identified using the single eluent approach, with a few new peptide sequences (Table 2). This result is consistent with the presence of a peptide-surface recognition mechanism, which is not sequence specific, but the result of complex interfacial interactions. The use of different phage libraries (Table 1) can also be responsible for the diversity of sequences isolated by the two methods. Sequence TVNFKLY appears as a frequent binder (8/30) in Gly-HCl pH 2.2. It is also the only sequence to be isolated from all three Gly-HCl eluents at varying pH values (Table S3). For TEA, three new sequences were identified (YSLKQYQ, YSFKQYQ, and YNGSANG) of similar pI to the pool isolated using the conventional panning approach. Sequence YSLKQYQ was isolated at both high and low pH elution conditions. The appearance of peptide sequences in the second and third steps of the elution process in a single round of panning shows that not all strong binders are eluted and recovered using the single elution approach with Gly-HCl pH 2.2. By using the sequential elution process, varying eluent type and/or pH conditions in a single panning round, the phage clones that resist detection in the Gly-HCl elution step are eluted in successive elution steps.

### Confirming Binding by Experimental/Computational Approaches

Binding experiments and bioinformatic tools were used to assess the authenticity of a selection of frequently isolated phage clones. The relative affinity assay has been reported as a simple and quick method to evaluate target binding ability of phage displayed sequence.^16−18^ Bioinformatic analysis was performed using the BDB database tools to screen for sequences identified as binders to other targets (TUPredict), sequences carrying TUP motifs (TUPScan), and fast growers (PhD7faster).

We used the relative binding affinity assay to assess the binding capability of each of 12 different phage clones by phage titer assays at pH 7 (Table S4). The selected sequences for these studies include nine phage clones frequently identified using conventional and optimized elution, and three phage clones (YNGSANG, ETALIAA, and GTGSQAS) isolated only once, as controls. Wild-type M13KE was also used as a further control to verify the binding of selected clones as a result of the randomly displayed peptide sequence and not due to nonspecific coat protein interactions. Of the frequently isolated clones, sequence TVNFKLY was excluded because it was the only sequence in this study to be isolated from all eluates, and due to its known ability to bind to other inorganic materials,^18^ and to polystyrene surfaces as confirmed by bioinformatic analysis.

**Figure 1:**
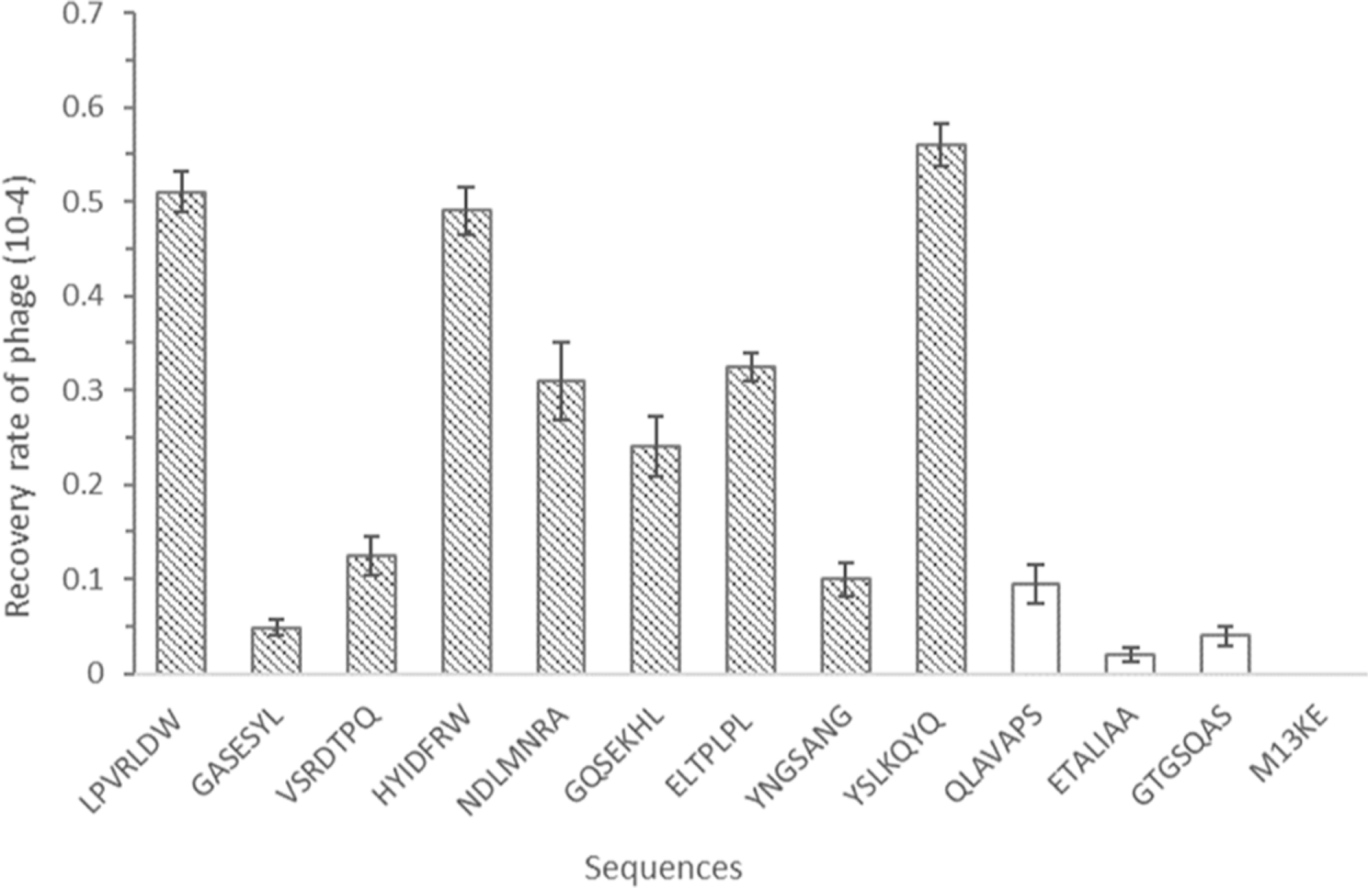
Binding ratio (bound/input) of each of the most frequently selected phage clones to silica nanoparticles. Patterned columns are frequently selected phage clones; white columns are phage clones isolated only once, as controls.

**Figure 2:**
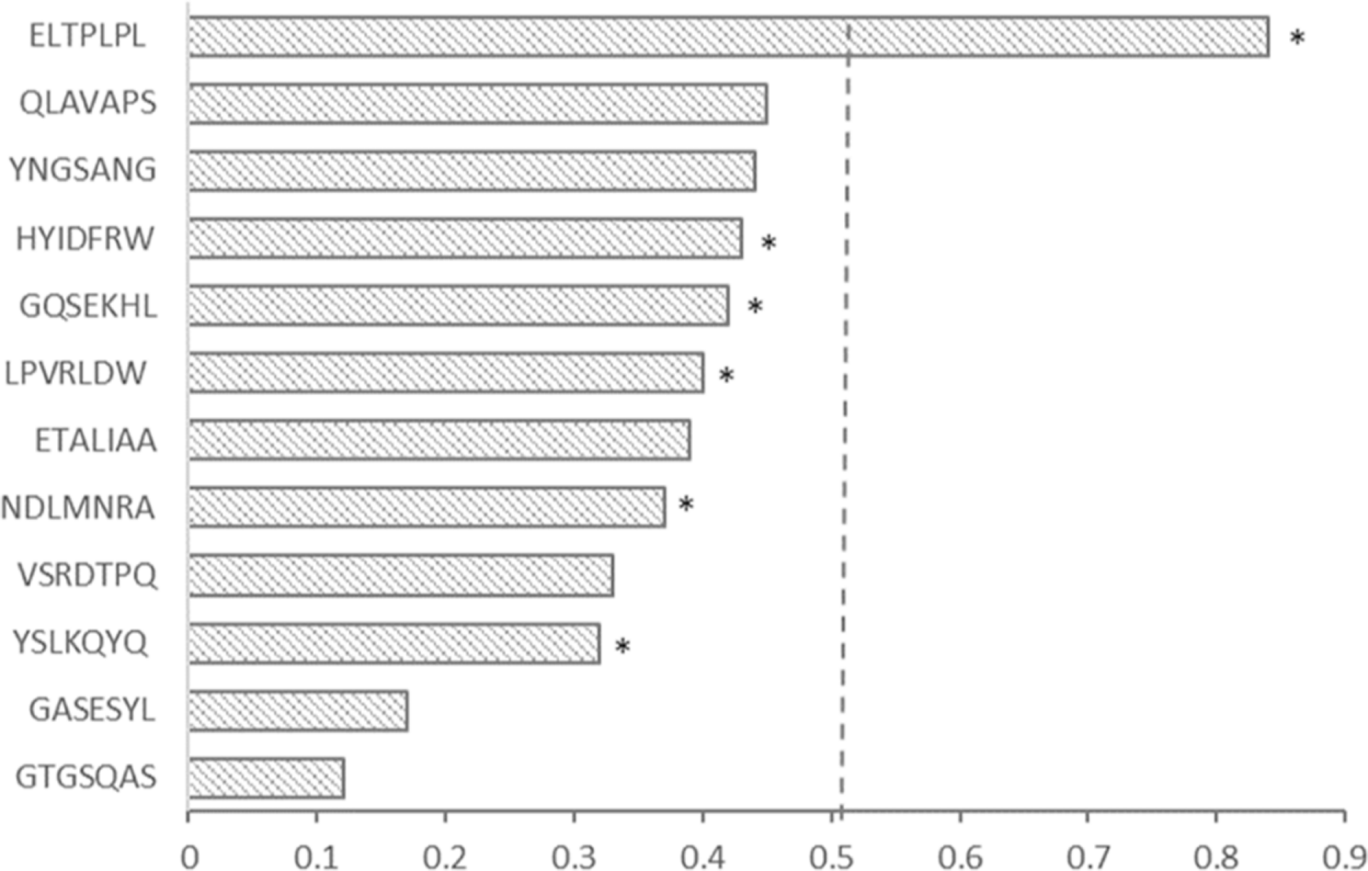
Prediction of the probabilities of target unrelated peptides or target binders. The probability values for silica binders were generated using the TUP predict tool (http://immunet.cn/bdb/index.php/site/tools?type=TUPredict) from the BDB database and plotted. An asterisk (*) indicates the sequences identified as tight binders to silica in experimental phage display and phage recovery experiments.

The relative affinity binding assay results show higher binding than the control M13KE for all selected clones, as shown in Figure 1. The phage clones displaying peptides YSLKQYQ (148 plaques), LPVRLDW (130 plaques), and HYIDFRW (126 plaques) emerged as the top three strong binders for amorphous silica nanoparticles followed by NDLMNRA (75 plaques) and ELTPLPL (77 plaques). The phage clones GQSEKHL, VSRDTPQ, and GASESYL although appearing more frequently in the panning experiments displayed lower affinity to silica in relative binding studies. Interestingly, the frequently isolated sequence ELTPLPL exhibited a higher binding ability to silica nanoparticles; however, it is the only sequence flagged as a fast grower by bioinformatic analysis using PhD7Faster predictor (Figure 2).

Bioinformatic analysis shows that none of the sequences identified in this work bear any known TUP motif. All frequently identified sequences except YSLKQYQ, LPVRDW, YNGSANG, and ELTPLPL appear to have been identified on other target materials according to the BDB database (Table S5), though the majority of these are molecular species as opposed to inorganic minerals. However, sequences QLAPAS, HYIDFRW, and GQSEKHL have been previously isolated as binders for Fe_3_O_4,_^18^ hence showing some level of promiscuity. This is similar to what observed for other well-known silica binders, like Pep1 (KSLSRHDHIHHH)^11^ which has also been isolated from panning against different TiO_2_ crystal surfaces^19^ and magnetite.^20^

### Peptide Selection for In Vitro Adsorption Binding Studies

We selected two peptide sequences: S4 (HYIDFRW) and S5 (YSLKQYQ) to investigate their binding on silica in vitro, and to study the effect of changing functionality at the C-terminal end from acid (COOH) to amide (CONH_2_) groups on the binding process. The sequences were synthesized and chemically modified by functionalizing the C-terminus with an amide group (details are given in the Supporting Information) and are designated S4–NH2 and S5–NH2. These sequences were selected based on their strong silica affinity, as determined by experimental and bioinformatic approaches; considering their physicochemical properties, type of interactions we predict will occur in vitro (Table 3). Peptide S4 (HYIDFRW) has a circumneutral pI and neutral charge at pH 7, while S5 (YSLKQYQ) is positively charged and has a more hydrophilic character. Both sequences were frequently identified in panning experiments and presented the highest phage recovery rates. Sequence LPVRLDW showed affinity; however, its physicochemical properties (pI: 6.85, net charge at pH7:0, ratio hydrophobic/hydrophilic residues: 29%) are similar to those of HYIDFRW and were therefore not selected for this study.

**Table 3:** Selection of Peptides Based on Experimental Phage Display Results, Physicochemical Properties, and Bioinformatic Analysis

criteria for peptide selection	HYIDFRW (S4)		YSLKQYQ (S5)	
Experimental/Bioinformatic				
panning method used	single eluent Gly-HCl (repanning)		optimized panning (Gly-HCl and TEA)	
relative binding result	high affinity binder		high affinity binder	
BDB probability of target binder	target binder (0.43)		target binder (0.32)	
BDB search results: (materials reported to have affinity for)	Fe_3_O_4_ NPs^18,21,22^		no hit	
Physico-Chemical/Interactions				
	OH	NH_2_	OH	NH_2_
pI value	7.83	9.85	9.60	10.11
net charge @7	+0.10	+1.10	+1.00	+2.00
ratio of hydrophilic/total number of residues	29%	57%		
likely type of interactions	○ Electrostatic		○ Electrostatic	
	○Hydrophobic		○H-bonding	
	○H-bonding			

However, it is common to select inorganic binding peptides merely relying on their in vivo phage display results, i.e., assuming the most frequently occurring peptides as the main binders^11^ often synthetic sequences display dissimilar in vitro binding behavior with nanoparticles even under similar conditions to those used in the phage display process. The complex surface chemistry of the nanoparticles and physicochemical properties of the peptides and the binding environment can contribute to these differences.^3,9,11^ By using a complementary approach, it is anticipated that the chosen peptides might show better binding behavior with silica nanoparticles under in vitro conditions than those selected only based on a single criterion (i.e., relying on experimental phage display data).

**Figure 3:**
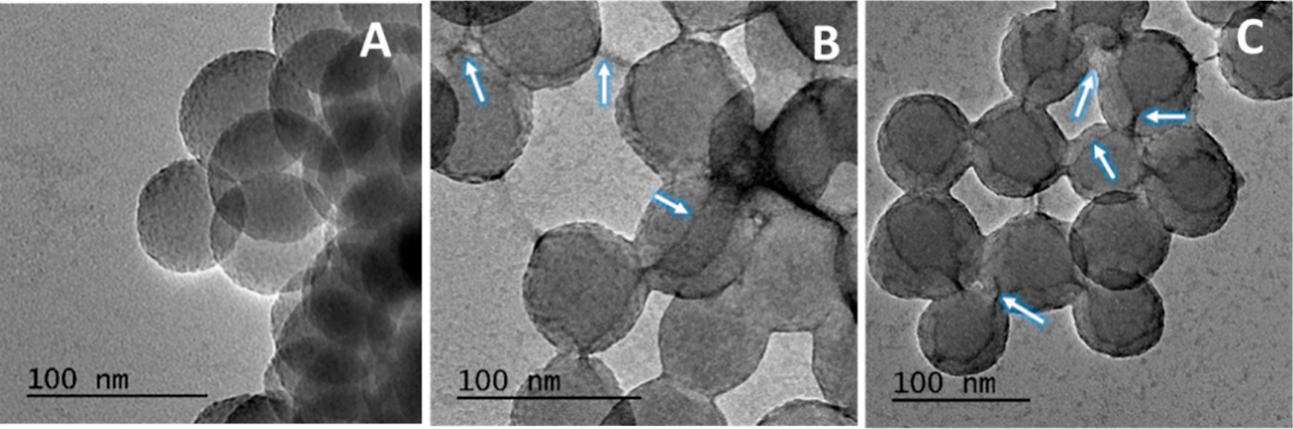
TEM images of (A) silica NPs (blank); (B) phage displaying sequence S4 (HYIDFRW); and (C) phage displaying sequence S5 (YSLKQYQ); phage structures thought to bind to silica are shown by white arrows.

We were able to further confirm binding of the phage clones, displaying each of the two sequences by TEM. A nonradioactive dye EM Stain 336 (Uranyl Acetate Alternative) was used to stain the peptide–silica complex affixed on TEM grids (see the Supporting Information for details). The uranyl type acetate ions present in the dye bind to peptides, which can be visualized under TEM conditions. Figure 3 shows how the phage clones displaying the two sequences bind to silica with data presented in Figure S1, showing the control images (silica + dye, phage + dye). The phage appears to be binding on the surface of silica by forming thread-like structures cross-linking adjacent silica particles, which can be seen indicated by white arrows. Furthermore, some phage structures seem to be folded and lie on the surface of the silica particles. It should be noted that the observed positioning of the phages on multiple silica particles may be an artifact of the sample preparation and drying procedure. In contrast, low-voltage TEM imaging of phages on clay shows binding phages as filaments attached to individual clay particles via one end.^23^ The use of Cryo electron microscopy would offer better understanding of the effective phage conformation and orientation upon binding to silica surface, similar to what has been carried out for cellular hosts.^24^

A fluorescamine assay^9^ was used to assess binding including the effect of C-terminus functionalization with an amide group to mimic peptide presentation to silica during phage display selection, where the peptide is attached to the phage protein via the C-terminus. Modification of the C terminus from acid to amide results in neutralization of the negative charge on the C end changes the net charge of the sequence (Table 3). It has been shown that resulting charge and conformational changes affect peptide binding affinity and have a direct impact on their biological activity.^25^

**Figure 4:**
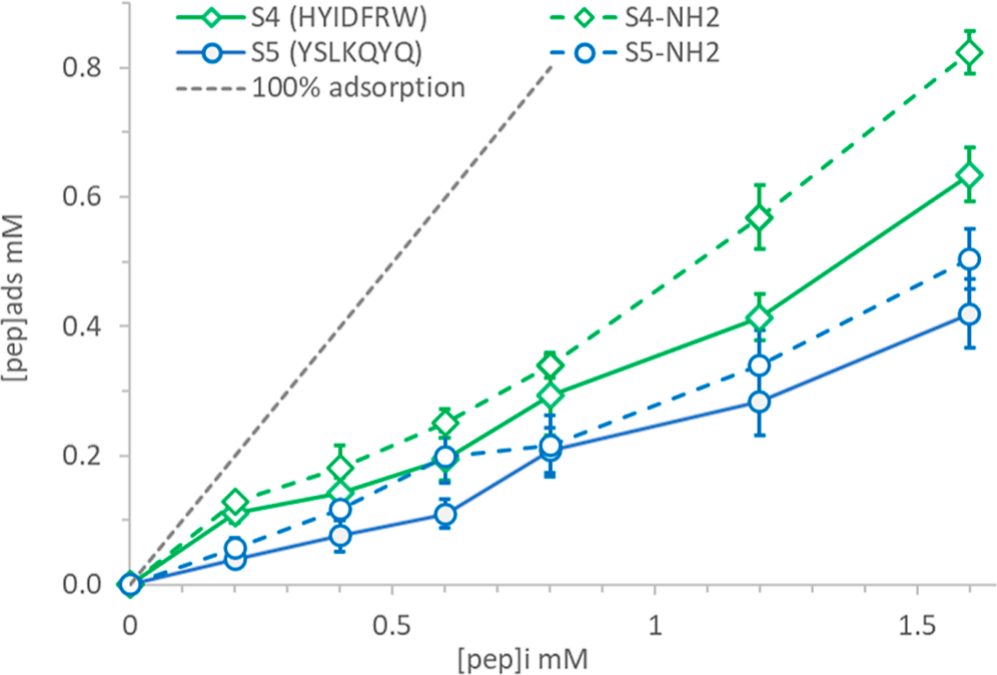
Adsorption isotherms of synthesized peptides with either acid (S4 and S5) or amide (S4–NH_2_ and S5-NH_2_) at the C-terminal group of selected phage displayed sequences showing their binding behavior on the surface of hydrophilic silica nanoparticles at pH-7.5. The *y*-axis represents the amount of peptide adsorbed to silica in mM, while the *x*-axis shows the initial peptide concentration or peptide added in mM. The error bars represent the standard deviation for triplicate analyses.

The absorption experiments confirmed in vitro high silica affinity of both sequences selected and their amide-functionalized version (Figure 4). Peptide S4 (HYIDFRW) is a better binder than S5 (YSLKQYQ) over the entire range of the initial peptide concentrations considered. The relative uptake of the two peptides shows a similar trend for initial peptide concentrations above 0.2 mM, with uptakes in the range 20–25% for S5 (YSLKQYQ) and 30–40% for S4 (HYIDFRW). However, at a low initial concentration (0.2 mM), the results show a significantly different binding behavior for the two peptides, with S4 (HYIDFRW) showing a relative uptake at 55%, almost three times higher than S5 (YSLKQYQ).

For S4 (HYIDFRW) which binds more at low concentrations and overall is a better binder across the whole concentration range, we can predict binding to derive from the contribution of several different forces, including a significant contribution from hydrophobic interactions and H bonding. The presence of basic amino acids His and Arg can drive binding via electrostatic interactions on the negatively charged silica surface. In addition, the polar uncharged amino acid Tyr along with charged polar residues including His and Arg can initiate binding via hydrogen bonding, resulting in peptide multilayers connected by hydrogen bonds.^9^ Finally, the three hydrophobic residues (isoleucine, aromatic phenylalanine, and tryptophan) and overall hydrophobic character of the sequence open up the possibility of hydrophobic interactions at the peptide–silica interface.^9^ In contrast, for S5 (YSLKQYQ) due to its high pI value and the presence of lysine, we can predict binding to be driven by electrostatic interactions, with a contribution from hydrogen bonding possible via the hydroxyl residues in Tyrosine and Serine.

The adsorption binding results show that for both peptides, the amide functionalization of the C-terminus resulted in higher binding to negatively charged silica nanoparticles compared to the corresponding carboxyl terminal peptides (Figure 4). Amide functionalization of the C-terminal has the effect of increasing the pI value of the peptides and an increase of one unit of net charge at pH 7.5, compared to the sequences with a carboxylic end.

Peptide S4 (HYIDFRW) shows the largest increase in binding affinity in the amide-functionalized sequence, especially at high initial peptide concentrations. This peptide is neutral at pH 7.5, while its amide-functionalized equivalent is positively charged +1, thus resulting in the strongest possible ionic interactions with the silica surface. The removal of the negative charge at the C-end reduces the repulsion with hydroxyl groups on the silica surface and allows for more hydrophobic interactions. The same amide group can act as a hydrogen bond acceptor and donor; whereby its presence can increase hydrogen bonding either with the silanol groups present on the silica surface; or with other peptide molecules forming peptide–peptide bonding resulting in multilayer formation; or both under these conditions. For peptide S5 (YSLKQYQ), where binding is driven by electrostatics and H bonding with no hydrophobic contribution, the benefit of reducing repulsion is less pronounced.

## Conclusions

In this work, we have built on our previous knowledge on in vitro peptide-silica binding to demonstrate how the use of Gly-HCl pH 2,2 does not remove all strong binders to a mineral such as silica. We show how the use of different eluents, or a single eluent at a wide range of pH values, can effectively allow the isolation of larger families of binding peptides of similar pI. We propose a novel sequential elution approach for the comprehensive isolation of separate families of inorganic binders in a single elution step. This is an important step toward the development of a more sustainable and time-efficient phage panning protocol We also show that peptides identified by phage display, once they are modified to account for the lack of a carboxyl terminus (as presented by the phage themselves), bind more strongly to the mineral phase highlighting the importance of the N-terminus in peptide recognition alongside H-bonding and hydrophobic interactions that tune peptide binding. This work provides with further understanding on how different eluents affect phage binder sequence identity, and thereby mode of interaction, giving the experimentalist control over aspects of molecular recognition which can be used to generate biocomposites. This may be instructive in the design and engineering of mineral-targeted constructs and composites for a range of biomedical and nanotechnological applications. Future work will aim to reduce amplification bias by using the optimized sequential approach over a single panning round, thereby minimizing the number of panning rounds required to identify target specific binders and better understand the physical chemical properties and binding behavior of S4 and S5 as a function of pH and binding environment.

## Supporting Information Available

Experimental details on phage panning protocols, peptide synthesis, relative binding affinity, and in vitro binding studies; TEM imaging; bioinformatic tools; complete list of isolates, phages, and their frequency; phage titer calculation; bioinformatic tool analysis; and TEM images of silica and M13 phage control samples (PDF)
