# Supplementary material for: Phage Display Panning on Silica: Optimization of Elution Conditions for Selection of Strong Binders
Source: Langmuir. 2024 Jul 17;40(30):15512–9. doi: 10.1021/acs.langmuir.4c01108 (PMC11295192; doi:10.1021/acs.langmuir.4c01108)
Supplement: Supplementary file 1 — la4c01108_si_001.pdf [file la4c01108_si_001.pdf]

Supporting Information

Phage display panning on silica: optimisation of elution conditions for selection of strong binders

*Veeranjaneyulu Thota, Valeria Puddu, Carole C. Perry**

Interdisciplinary Biomedical Research Centre, School of Science and Technology,

Nottingham Trent University, Clifton Lane, Nottingham NG11 8NS, U.K.

*Carole.Perry@ntu.ac.uk

Number of pages 9

Number of figures 1

Number of tables 5

EXPERIMENTAL DETAILS:

1. Panning protocols.

The target material was a batch of amorphous silica nanoparticles 82 ± 4 nm in size that have been previously fully characterised and used in multiple studies of peptide binding.[1-3] All panning experiments were done using 1 mg of SiO_2 particles. Prior to panning the silica was washed three times with 1 ml 0.3% TBST (0.1% Tween 20 [v/v] in 1ml of TBS), vortex rotated for 3 min and recovered by centrifugation at 12,000 rpm, to remove any impurities.
The Ph.D.7 phage display peptide library kit (#E8100S batch 0211212) was purchased from New England Biolabs (NEB).

10 µl of the Ph.D.-7 library containing 1 x 10^{13} pfu/ml was exposed to 1 mg/ ml of silica nanoparticles in TBS buffer at pH-7.5 and incubated for 60 minutes.

The library manufacturer's instructios were followed for all amplification and titering procedures. Changes to the manufacturer's instructions were applied to washing, elution and neutralisation steps to obtain the three panning protocols below.

1.1 Conventional panning: after binding, the silica was washed 10 times with 1ml of 0.5% Tween 20 in TBS. Three different eluents with varying pH (0.2 M Glycine-HCl, pH-2.2; 4 M $MgCl_2$, pH-6.1; 0.01 M TEA, pH-11) were tested to elute and collect strongly bound phage. Gly-HCl eluates were neutralised with 150 µl of 1 M Tris HCl at pH-9.1, while 4 M $MgCl_2$ and triethylamine eluates were neutralised by adding 50 µl of 1 M Tris HCl at pH 9.1 or 150 µl of 1 M Tris HCl at pH 5, respectively prior to amplification.

For each of the three different eluents, up to 3 rounds of panning were performed before titering and plaque selection via the white-blue screening method. Selected plaques were then amplified and sequenced.

1.2 Repanning experiments: performed to cross check the reproducibility of the identified phage displayed sequences. In this process 50 µl of the amplified phage pool obtained from round 2 of separate conventional panning experiments with the three different elutents were mixed together and named 'mix of amplified phage'. This mix was then repanned against fresh silica, followed by 10 washes with TBST 0.5% - 0.7% in successive rounds, eluted at low and high pH values (Gly-HCl pH 2.2 and TEA pH 11, respectively) and sequenced at rounds 3 for Gly-HCl pH 2.2; and at round 4 for TEA pH 11 (see table SI-2).

1.3 Optimised 'three step' panning: after binding, the silica was washed with 0.5% - 0.9% Tween 20 in TBS (5 washes each). In the second round of panning the target was washed with 0.3 and 0.5% TBST (5 washes each).

Two alternative elution-based approaches were tested. In the first approach, three different eluents - i.e: 0.2M Gly-HCl, pH-2.2; 4M $MgCl_2$, pH-6.1; 0.1M TEA, pH-11 - were used consecutively at each elution step of a panning round. In the second approach, 0.2M Gly-HCl solution at different pH (pH-2.2, pH-7, pH-11) was used consecutively for the elution step. Eluted phage clones were screened after three rounds of panning following manufacturer's instructions.

2. Binding studies:

2.1 Peptide synthesis: Peptides were synthesized using an automated single channel microwave assisted peptide synthesizer (CEM Liberty1) following standard Fmoc chemistry and starting from preloaded Wang resins as solid supports to initiate the peptide synthesis. The amide derivatives were prepared using Fmoc-Rink Amide ProTide Resins. These synthesized peptides were characterized by HPLC and mass spectrometry. Peptide purity by HPLC was in the range 78 to 94%.

2.2 *In vitro* silica binding studies: suspensions of silica nanoparticles (1 mg/ ml) in phosphate-buffered saline buffer were sonicated for 1 hour, then peptide was added to achieve the desired initial peptide concentration in the range 0.2-1.6 mM. The mixtures were then shaken vigorously and left to equilibrate for 1 hour at room temperature. The silica was separated by centrifugation (12,000 rpm for 5 min) and the supernatant was analysed by fluorimetric assay to quantify the amount of peptide left in solution.[2] The amount of peptide bound was then calculated by difference.

2.3 Relative binding affinity (phage binding assay): A selection of 15 clones were individually amplified and screened against fresh silica. Silica (1mg) was washed 10 times with TBST (0.3% TWEEN [v/v] in 1 ml TBS) at pH 7.5 then incubated with 0.01 ml of 2.5E+10 pfu/ml of phage clones (input phage), and binding affinity determined via titer assay, and calculated as the ratio of output phages to input phages. Dilution factor was 10E+04 for all peptide displaying clones, and 10E+03 for wild MK13 control.

$$Bound\ phage\left(\frac{pfu}{ml}\right) = \frac{number\ of\ phage\ plaques}{volume} \times (dilution\ factor) \qquad \text{equation S1}$$

2.4 Transmission electron microscopy (TEM) imaging: The amplified phage clones displaying each of the selected peptides YSLKQYQ and ADIRHIK were mixed with silica nanoparticles suspensions (1mg/ml) and incubated on either Poly-L-lysine and/ or paraformaldehyde coated TEM grids. The grid containing silica-phage suspension was washed three times with distilled water to remove weakly bound phage and stained with a drop (roughly 5 μl) of diluted aqueous

EM stain 336 (Agar Scientific) then left for 30 min at room temperature. The grids were then air dried before washing three times with distilled water (roughly 5 µl each wash). The air-dried samples were then visualised using a JEOL 2010 TEM at 200 KeV using a LaB_6 filament to confirm phage-silica binding. For comparison, silica alone, silica with the dye and a drop of the Ph.D.™-7 phage display peptide library (New England Biolabs) with dye was observed under TEM. Control data for silica + dye and phage + dye is shown in Figure S1.

3. Bioinformatics

Sequences were analysed by tools available on the BDB databank. [4] These tools were developed using machine learning algorithms and three predictors namely PhD7Faster, SABinder and PSBinder were employed. The PhD7Faster tool was used to predict if phages bearing randomly displayed peptides from the Ph.D.-7 library might grow faster due to propagation advantage. [5] The silica binders identified in this study were checked by entering the sequence data either in FASTA or raw sequence data format. The threshold to distinguish between predicted positives and negatives (*tp*) was set to 0.5 with a peptide being predicted to be a target unrelated peptide (TUPs) if the probability is 0.5 or higher.

EXPERIMENTAL DATA

Table S1. Complete list of sequences isolated from conventional panning experiments after three rounds of panning. [a]pI values were obtained from Bachem peptide calculator (http://www.bachem.com/)

Eluent	TBST Wash	Round screened	Sequence	pI[a]	frequency	Frequently observed sequence
Gly-HCl pH 2.2	0.5%	3	LPVRLDW	6.85	25/25	LPVRLDW(25)
	0.5%	3	GASESYL	3.27	6/20	
			VSRDTPQ	6.85	3/20	
			QQTNWSL	6.01	2/20	
			WQWPARV	11.06	2/20	
			NDLMNRA	6.85	1/20	
			GQSEKHL	7.88	1/20	
			QLAVAPS	6.01	1/20	
			ETALIAA	3.27	1/20	

			GTGSQAS<br>ALQPQKH<br>VASHSKP	6.01<br>10.13<br>10.13	1/20<br>1/20<br>1/20	GQSEKHL(10)<br>GASESYL (6)<br>NDLMNRA (6)<br>VSRDTPQ (4)
$MgCl_2$	0.5%	3	LPVRLDW<br>NDLMNRA<br>GQSEKHL<br>QQTNWSL<br>VGSYLGI<br>QLAVAPS	6.85<br>6.85<br>7.88<br>6.01<br>5.97<br>6.01	5/14<br>4/14<br>2/14<br>1/14<br>1/14<br>1/14	
TEA	0.5%	3	GQSEKHL<br>ELTPLPL<br>NDLMNRA<br>VSRDTPQ<br>KIAVIST<br>QHMPQPR	7.88<br>3.27<br>6.85<br>6.85<br>10.13<br>11.06	7/13<br>2/13<br>1/13<br>1/13<br>1/13<br>1/13	

Table S2. Complete list of sequences isolated from repanning experiments showing recurrence of silica binders previously identified after three rounds of conventional panning. [a]pI values were obtained from Bachem peptide calculator (http://www.bachem.com/)

			**Previous binders**		**New Binders**	
**Eluent**	**TSBT wash**	**Round**	**Sequences (frequency)**	**pI[a]**	**Sequence (frequency)**	**pI[a]**
Gly-HCl pH 2.2	0.5%	3	KIAVIST (7/30)<br>QLAVAPS (3/30)<br>ETALIAA (2/30)<br>GQSEKHL (2/30)<br>VSRDTPQ (1/30)<br>LPVRLDW (1/30)<br>ELTPLPL (1/30)	10.13<br>6.01<br>3.27<br>7.88<br>6.85<br>6.85<br>3.27	HYIDFRW (2/30)<br>HVPRAMA (1/30)	7.83<br>11.06
$MgCl_2$	0.7%	4	KIAVIST (10/30)<br>VSRDTPQ (7/30)<br>LPVRLDW (2/30)<br>GQSEKHL (1/30)<br>ELTPLPL (1/30)<br>QQTNWSL (1/30)<br>GTGSQAS (1/30)	10.13<br>6.85<br>6.85<br>7.88<br>3.27<br>6.01<br>6.01	HVPRAMA (4/30)<br>HYIDFRW (1/30)<br>SQTFTSD (1/30)	11.06<br>7.83<br>3.09
TEA	0.7%	4	KIAVIST (12/30)<br>GQSEKHL (4/30)	10.13<br>7.88	HYIDFRW (3/30)<br>SFPLSKY (3/30)	7.83<br>9.67

			ELTPLPL (3/30)<br>VSRDTPQ (1/30)<br>NDLMNRA (1/30)<br>QQTNWSL (1/30)	3.27<br>6.85<br>6.85<br>6.01	TVNFKLY (1/30)<br>HGGVRLY (1/30)	9.67<br>9.85

Table S3. Experimental results of optimised sequential panning approach using Gly-HCl at different pH values. In bold are indicated frequently identified silica binders.

Gly-HCl pH-2.2	Frequency	Gly-HCl pH-7	Frequency	Gly-HCl pH-11	Frequency
**VSRDTPQ**	5/30	**VSRDTPQ**	6/30	**VSRDTPQ**	6/30
**TVNFKLY**	4/30	**TVNFKLY**	3/30	**TVNFKLY**	3/30
**KIAVIST**	2/30	-	-	-	-
**HGGVRLY**	2/30	-	-	-	-
GQSEKHL	1/30	**GQSEKHL**	3/30	GQSEKHL	1/30
-	-	**YSLKQYQ**	3/30	YSLKQYQ	1/30
-	-	**ELTPLTL**	2/30	ELTPLTL	1/30
LPVRLDW	1/30	LPVRLDW	1/30	-	-
WSLSELH	1/30	-	-	WSLSELH	1/30
-	-	NDLMNRA	1/30	-	-
**QQTNWSL**	2/30	QQTNWSL	1/30	-	-
FASRSDT	1/30	-	-	FASRSDT	1/30
		YNGSANQ	1/30		

		TTQVLEA	1/30	TTQVLEA	1/30
		VKMESPL	1/30	VKMESPL	1/30
		SWTALGP	1/30		
		QMHREPA	1/30	QMHREPA	1/30
QSMPQAR	1/30	-	-	ENHVHVR	1/30
NQIYSAN	1/30	-	-	VENVHVR	1/30
STPATLI	1/30	-	-	VASHSKP	1/30
NAPYRAM	1/30	-	-	VAPPRLI	1/30
WTTSVGT	1/30	-	-	GQGQTIP	1/30
TNLSHVP	1/30	-	-	DSMSLLQ	1/30
VHRDSWT	1/30	-	-	AHINVPS	1/30
-	-	VVTPKTA	1/30	SFNPLAY	1/30
Nil	6/30	Nil	4/30	Nil	7/30

Table S4. Phage titer calculations for relative binding assay. Experiment was run twice. Bound phage was calculated from equation SI-1. Input phage is 2.5E+05

	Experiment 1			Experiment 2		
	Number of plaques obtained	bound phage (pfu/ml)	Binding ratio (bound/input *100)	Number of plaques obtained	bound phage (pfu/ml)	Binding ratio (bound/input *100)
LPVRLDW	130	1.3E+08	0.52	123	1.2E+08	0.492
GASESYL	12	1.2E+07	0.048	14	1.4E+07	0.056
VSRDTPQ	33	3.3E+07	0.132	27	2.7E+07	0.108
HYIDFRW	126	1.3E+08	0.504	116	1.2E+08	0.464
NDLMNRA	75	7.5E+07	0.3	89	8.9E+07	0.356
GQSEKHL	54	5.4E+07	0.216	63	6.3E+07	0.252
ELTPLPL	77	7.7E+07	0.308	85	8.5E+07	0.34
YNGSANG	29	2.9E+07	0.116	23	2.3E+07	0.104
YSLKQYQ	148	1.5E+08	0.592	136	1.4E+08	0.568
QLAVAPS	19	1.9E+07	0.076	26	2.6E+07	0.104
ETALIAA	2	2.0E+06	0.008	7	7.0E+06	0.028

GTGSQAS	9	9.0E+06	0.036	16	1.6E+07	0.064
M13KE	23	2.3E+06	0.0092	13	1.3 E+06	0.0052

Table S5. Mimo search/ scan/ Blast analysis results of silica binders identified. Frequently identified silica binders that showed hits for other target materials indicating that they might be promiscuous binders.

Peptide	Target Found	Matched Sequence	Library type	Panning round
QLAVAPS<br>TVNFKLY	Fe_3O_4 Nanoparticles [6]	Full sequence	Ph.D.-7	4, 5
HYIDFRW	Fe_3O_4 Nanoparticles [6-8]	"	Ph.D.-7	2-5
GQSEKHL	Fe_3O_4 Nanoparticles<br>Sera of peanut-tolerant subject [8]	"	Ph.D.-7	2-5
VSRDTPQ<br>ETALIAA<br>FASRSDT	Sera of peanut allergic and patients [8]	"	Ph.D.-7	1-3
GASESYL	Sera of peanut allergic patient [8, 9]<br>Anti-TIM polyclonal antibody [9]	"	Ph.D.-7	1-3

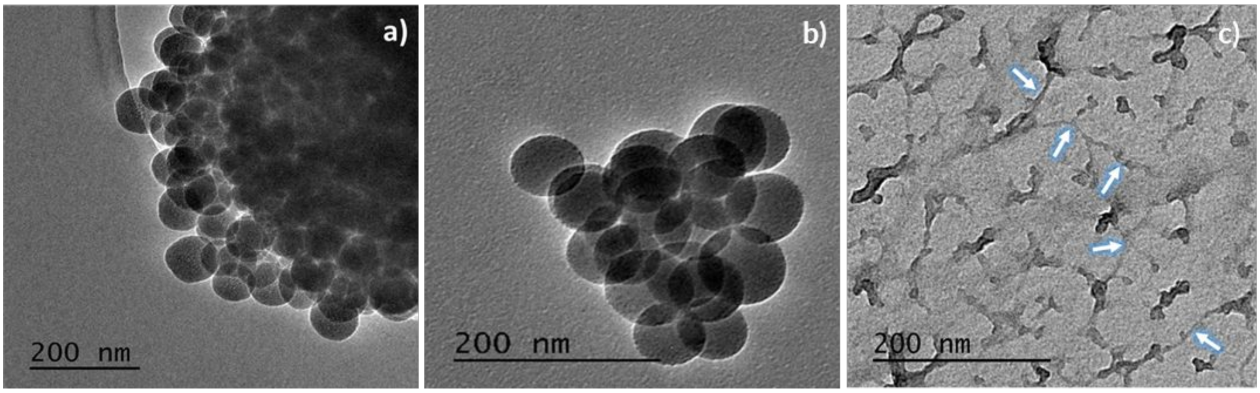

Figure S1. Control TEM images of a) silica NPs (82nm) without EM stain 336; b) silica NPs with EM stain 336; c) M13 Ph.D.7 phage library stained with EM stain 336. Phage structures are indicated by white arrows.

REFERENCES

1. Puddu, V.; Perry, C. C. Interactions at the Silica-Peptide Interface: The Influence of Particle Size and Surface Functionality. *Langmuir* 2014*, 30*, 227-233.

2. Puddu, V.; Perry, C. C. Peptide Adsorption on Silica Nanoparticles: Evidence of Hydrophobic Interactions. *ACS Nano* 2012*, 6*, 6356-6363.

3. Patwardhan, S. V.; Emami, F. S.; Berry, R. J.; Jones, S. E.; Naik, R. R.; Deschaume, O.; Heinz, H.; Perry, C. C. Chemistry of Aqueous Silica Nanoparticle Surfaces and the Mechanism of Selective Peptide Adsorption. *J. Am. Chem. Soc.* 2012*, 134*, 6244-6256.

4. He, B., et al BDB: Biopanning Data Bank. *Nucleic Acids Res* 2016*, 44*, D1127-D1132.

5. Zade, H. M.; Keshavarz, R.; Shekarabi, H. S. Z.; Bakhshinejad, B. Biased Selection of Propagation-Related TUPs from Phage Display Peptide Libraries. *Amino Acids* 2017*, 49*, 1293-1308.

6. You, F.; Yin, G.; Pu, X.; Li, Y.; Hu, Y.; Huang, Z.; Liao, X.; Yao, Y.; Chen, X. Biopanning and Characterization of Peptides with Fe3O4 Nanoparticles-Binding Capability Via Phage Display Random Peptide Library Technique. *Colloids Surf. B Biointerfaces* 2016*, 141*, 537-545.

7. Bashari, O.; Redko, B.; Cohen, A.; Luboshits, G.; Gellerman, G.; Firer, M. A. Discovery of Peptide Drug Carrier Candidates for Targeted Multi-Drug Delivery into Prostate Cancer Cells. *Cancer Lett.* 2017*, 408*, 164-173.

8. Christiansen, A.; Kringelum, J. V.; Hansen, C. S.; Bogh, K. L.; Sullivan, E.; Patel, J.; Rigby, N. M.; Eiwegger, T.; Szepfalusi, Z.; de Masi, F.; Nielsen, M.; Lund, O.; Dufva, M. High-Throughput Sequencing Enhanced Phage Display Enables the Identification of Patient-Specific Epitope Motifs in Serum. *Sci. Rep.* 2015*, 5*, 12913.

9. Yang, Y.; Chen, Z.; Hurlburt, B. K.; Li, G.; Zhang, Y.; Fei, D.; Shen, H.; Cao, M.; Liu, G. Identification of Triosephosphate Isomerase as a Novel Allergen in Octopus Fangsiao. *Mol. Immunol.* **2017***, 85*, 35-46.
